# Acute coronary syndromes did not decrease during COVID-19 pandemic in an isolated Greek population

**DOI:** 10.21542/gcsp.2024.39

**Published:** 2024-08-01

**Authors:** Matthaios Didagelos, Dimitrios Afendoulis, Eleni Karlafti, Maria Moutafi, Dimitrios Tsavdaris, Petros Voutas, Stefanos Garoufalis, Nikolaos Papagiannis, Nikolaos Smyrnioudis, Antonios Ziakas, Athanasios Kartalis

**Affiliations:** 1 1^*st*^ Cardiology Department, AHEPA University General Hospital, Thessaloniki, Greece; 2 Cardiology Department, “Skylitseio” General Hospital of Chios, Greece; 3 Emergency Department, AHEPA University General Hospital, Thessaloniki, Greece

## Abstract

**Introduction:** There are worldwide reports that hospital admissions for acute coronary syndrome (ACS) have declined during the COVID-19 pandemic. Chios is a Greek island with only one confirmed coronavirus case during the lockdown. This study aimed to compare admissions for ACS in Chios General Hospital, Greece, between the COVID-19 lockdown period and the same period in the previous year.

**Methods:** Retrospective record analysis of an isolated insular population referring to the only district hospital on the island. ACS incidence, type, and complications were recorded and compared between 26/02/2020–04/05/2020 and between 26/02/2019–04/05/2019.

**Results:** ACS hospital admissions in 2020 were 1.72/10,000 inhabitants *vs.* 1.03/10,000 inhabitants in 2019 (*p* = 0.317). No differences in ACS type, duration from symptom onset to first medical contact, hemodynamic status, left ventricular function at discharge, or complications were recorded.

**Conclusion:** The incidence of ACS did not decrease and the prognosis was not worse during the COVID-19 pandemic in a strictly isolated Greek insular population not overwhelmed by coronavirus cases.

## Introduction

The COVID-19 pandemic has changed the way people think and behave globally. Medical practice is trying to evolve to face new challenges while moving forward into uncharted routes. Lockdown policies were applied by almost every government in an attempt to limit the spread of coronavirus. Additionally, most health system resources and attention have focused on the fight against the coronavirus. From a cardiology perspective, there are worldwide reports that hospital admissions for acute coronary syndromes (ACS) declined during this period^1–3^.

Chios is the sixth largest Greek island (in terms of population), located in the Northeastern Aegean, with a stable population of 53,000 residents and approximately 5,000 immigrants (since 2015). The advantage of performing population studies on this island is that there is only one district hospital that hospitalizes all cases of ACS. This advantage was utilized in our previous report comparing ACS between two periods including the Greek socio-economic crisis in 2008^4^.

The aim of this study was to compare admissions for ACS in Chios General Hospital between the COVID-19 lockdown period and the same period of the previous year.

## Methods

### Study design

We retrospectively analyzed the Chios General Hospital Emergency Department and admission records for ACS, including ST-elevation myocardial infarction (STEMI) and non-ST-elevation myocardial infarction (NSTEMI). Cath lab records, disease course, and cardiology discharge letters were also reviewed to obtain details about ACS characteristics and complications (ventricular tachycardia/ventricular fibrillation requiring cardioversion, acute pulmonary edema, and death).

ACS was diagnosed according to the current guidelines of the European Society of Cardiology and was based on the clinical presentation, electrocardiographic (ECG) changes, and laboratory findings. Specifically, clinical presentations that support the diagnosis of ACS include various typical symptoms such as chest pain or discomfort (which can radiate to the jaw, neck, or arm) with accompanying symptoms such as dyspnea, diaphoresis, and nausea, or atypical symptoms more common in elderly, diabetic, and female patients^5^.

The ECG change suggesting a STEMI diagnosis is the new ST-segment elevation at the J point in at least two contiguous leads:

• ≥2.5 mm in men <40 years, ≥2 mm in men ≥40 years, or ≥1.5 mm in women regardless of age in leads V2–V3
• and/or ≥1 mm in the other leads (in the absence of left ventricular hypertrophy or left bundle branch block [LBBB]).

NSTEMI was diagnosed in patients without new ST-segment elevation but with ST-segment depression, T-wave inversion, normal ECG, or other ECG changes indicative of myocardial ischemia, but with symptoms suggestive of myocardial ischemia and elevated troponins. Based on laboratory findings, the quantification of troponins plays an important role. Elevated troponin levels above the 99th percentile of the upper reference limit confirmed a myocardial injury. Finally, in the diagnosis, it can also advocate various typical symptoms such as chest pain or discomfort, which could radiate to the jaw, neck, or arm, with accompanying symptoms such as dyspnoea, diaphoresis, and nausea or atypical symptoms more common in the elderly, diabetic, and female patients^5^.

### Study periods

Period 1: From February 26, 2020 (first confirmed COVID-19 case in Greece) until 04 May, 2020 (gradual release of strict lockdown). Period 2: The same dates during the year 2019.

### Statistical analysis

Categorical variables were compared using the chi-squared test. Continuous variables were compared using the *t*-test or Mann–Whitney U test if they were normally or non-normally distributed, respectively. Statistical significance was defined as *p* < 0.05. The analysis was performed using the IBM SPSS Statistics version 23 software.

The incidence rate of ACS was the primary outcome of statistical analysis. We hypothesized that there would be a high reduction in the incidence of ACS during the COVID-19 period. To detect this difference with an alpha level of 0.05, we calculated the necessary sample size. The estimated sample size was 32 patients out of approximately 53,000 permanent residents of the island. This calculation accounted for the expected variation in ACS incidence and ensured that the study could detect statistically significant differences between the two periods despite the relatively small absolute number of cases.

## Results

A total of 32 patients were admitted to the Chios General Hospital for ACS during the two periods (Table 1). ACS patients in the 2020 COVID-19 period were older, non-smokers, and had a positive family history of coronary artery disease.

**Table 1:** ACS characteristics between the two periods.

	2020 (*n* = 20)	2019 (*n* = 12)	*P* value
Baseline characteristics			
Age, years	73 (69–78)	53 (48–67)	0.002
Females	6 (30)	2 (16.7)	0.68
Males	14 (70)	10 (83.3)	0.68
Arterial Hypertension	16 (80)	6 (50)	0.12
Dyslipidemia	12 (60)	8 (66.7)	1.0
Diabetes	10 (50)	2 (16.7)	0.08
Smoking	8 (40)	12 (100)	0.001
Family History CAD	6 (30)	0	0.06
Previous History CAD	6 (30)	2 (16.7)	0.68
ACS details			
ACS incidence, %	1.72/10 000	1.03/10 000	0.32
STEMI	4 (20)	6 (50)	0.12
NSTEMI	16 (80)	6 (50)	0.12
Symptom onset, hours	3.3 (2.5)	1.8 (1.1)	0.08
Hemodynamically stable	16 (80)	8 (66.7)	0.43
Cardiogenic shock	4 (20)	4 (33.3)	0.60
Angiogram performed	20 (100)	8 (66.7)	0.01
Only medical management	6 (30)	6 (50)	0.29
LVEF on discharge, %	53 (6)	56 (4)	0.14
Complications	6 (30)	4 (33.3)	1.0
Death (%)	0	4 (33.3)	0.01

Notes.

Categorical data are expressed as n (%).

Continuous data are expressed as mean (SD) or as median (25th–75th percentiles).

ACS acute coronary syndromes SD standard deviation CAD coronary artery disease STEMI ST-elevation myocardial infarction NSTEMI Non-ST-elevation myocardial infarction h hours LVEF left ventricular ejection fraction

The incidence rate of ACS in the 2020 period was 1.72 per 10,000 inhabitants, with a 95% confidence interval (CI) of 1.0 2.4. For the 2019 period, the incidence rate was 1.03 per 10,000 inhabitants, with a 95% CI of 0.6 to 1.5.

**Figure 1.**
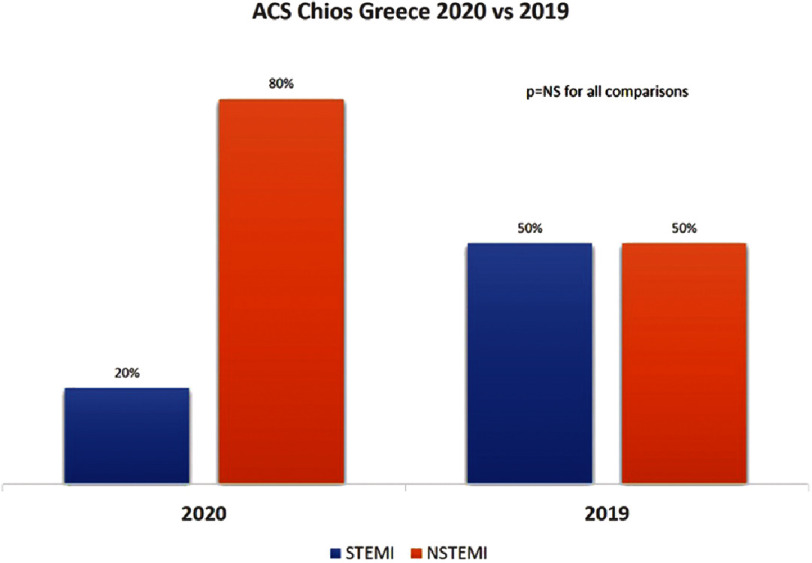
Type of ACS and relative incidence between the two study periods. ACS, acute coronary syndromes; NS, not statistically significant; STEMI, ST-elevation myocardial infarction; NSTEMI, Non-ST-elevation myocardial infarction.

Although total ACS admissions were numerically higher in the COVID-19 period (20 *vs.* 12 patients), the incidence of ACS was not statistically significant between the two periods (1.72/10,000 *vs.* 1.03/10,000 inhabitants, *p* = 0.317). There was a trend towards an increase in NSTEMIs *vs.* STEMIs between the two periods (80%/20% in 2020 *vs.* 50%/50% in 2019), although it did not reach statistical significance (*p* = 0.076), probably due to the small sample size. Regarding ACS characteristics between the two periods, no statistical differences were found in the duration from symptom onset to first medical contact, hemodynamic status, left ventricular function at discharge, and complications. (Table 1, Figure 1).

## Discussion

During this unprecedented time, reports from China, Italy, and Austria have shown a decrease in hospital admissions for ACS with a concomitant increase in the time from symptom onset to first medical contact and subsequent increased complications and mortality, when compared with a relevant period before the pandemic^1–3^. This has been attributed mainly to a delayed patient or system response because of the lockdown and misinterpretation of symptoms, such as chest pain or dyspnea, which are also related to COVID-19 infection.

However, in our isolated insular population, the total incidence of hospital admissions for ACS did not decrease. No increase in the time taken to seek medical contact or complications was recorded.

In all the aforementioned countries, COVID-19 cases were higher than in Greece, although all of them applied lockdown measures at some point. However, Greece was one of the countries that applied very early and harsh lockdown measures for a long period. All shops and schools were closed immediately, and restrictions on people’s movements were enforced and controlled by the police. Another measure was to completely isolate all Greek islands with the cancellation of all flight and ship connections to the mainland, with no people allowed in or out. Fortunately, Chios Island had only one confirmed coronavirus case during this period. This fact probably affected local society’s psychology, who did not appear afraid of seeking medical help and visiting the hospital, and did not lead to worse outcomes for ACS patients during the COVID-19 pandemic.

The findings of this study have many implications for clinical practice, especially regarding other isolated or low COVID-19 incidence areas. In particular, the results suggest that in areas with a robust healthcare system and minimal impact of COVID-19, the provision of emergency cardiovascular care can be effectively maintained during a pandemic. Strict and timely measures in Greece proved effective in controlling the spread of COVID-19 while maintaining access to basic health services. Other regions with similar geographic and demographic characteristics may benefit from similar strategies to control the pandemic with ongoing healthcare needs.

The main advantages of this study are the well-defined insular population, which was further isolated during the pandemic period, and the only district hospital on the island that allows for ACS cases to be completely traced and recorded. Limitations include the retrospective analysis of patient records and the small absolute numbers of ACS cases. Another limitation of this study is the lack of long-term patient data due to the lack of follow-up. There are also several factors that confound the findings of this study. During the study period, changes in healthcare-seeking behaviors could have been influenced by factors other than COVID-19, such as seasonal variations in illness patterns or modifications in local healthcare policies. While the strict lockdown measures and minimal COVID-19 incidence in Chios likely influenced the results, other societal factors unrelated to the pandemic could have contributed to the observed trends.

## Conclusion

The incidence of ACS did not decrease and the prognosis was not worse during the COVID-19 pandemic in a strictly isolated Greek insular population not overwhelmed by coronavirus cases.

## Highlights

1. Hospital admissions for acute coronary syndromes seem to decline during the COVID-19 pandemic period, in countries with large numbers of confirmed cases.
2. Chios is a Greek island located in the Northeastern Aegean, with a stable population and only one district hospital that hospitalized all cases of ACS. It was completely isolated during the COVID-19 lockdown period in Greece. Only one COVID-19 case was confirmed during this period.
3. Hospital admissions for acute coronary syndromes in Chios did not decrease during the COVID-19 period and complications were not increased.
4. The single COVID-19 case probably did not negatively affect the local society’s psychology, which was not concerned with seeking medical help and visiting the hospital.

## Statement

All authors take responsibility for all aspects of the reliability and freedom from bias of the data presented and their discussed interpretations.

## Conflicts of interest

None by all authors.

## Funding

This research received no specific grant from any funding agency in the public, commercial, or not-for-profit sectors.

## Ethical approval

Not applicable.

## Informed Consent

Not applicable.

## Trial Registration

Not applicable.
